# Design and Evaluation of Web-Based Dental Implant Registry (DIR) for Better Clinical Outcomes

**DOI:** 10.1155/2022/7162645

**Published:** 2022-02-11

**Authors:** Roya Naemi, Majid Jangi, Hamid Reza Barikani, Leila Shahmoradi

**Affiliations:** ^1^Department of Health Information Management, School of Paramedical Sciences, Ardabil University of Medical Sciences, Ardabil, Iran; ^2^Health Information Technology Research Center, Isfahan University of Medical Sciences, Isfahan, Iran; ^3^Dental Implant Research Center, Dental Research Institute, Tehran University of Medical Sciences, Tehran, Iran; ^4^Department of Health Information Management, School of Allied Medical Sciences, Tehran University of Medical Sciences (TUMS), Tehran, Iran

## Abstract

**Introduction:**

Identification of dental implant system in undocumented patients is a major challenge for dentists due to the vast variety of tools and technologies that are used in dental care. It also takes a long time to identify the type of connection or length and diameter of implant. To obtain accurate and timely information, it is necessary to have a Dental Implant Registry (DIR). In the present study, a DIR was designed, developed, and evaluated at the Dental Implant Research Center of Tehran University of Medical Sciences.

**Materials and Methods:**

This is an applied, developmental, and cross-sectional study that was conducted between 2018 and 2020. In the present study, after determining the objectives of DIR system, its conceptual model was designed by EDraw Max 7.9 software. Then, the registry was developed in Visual Studio 2018 environment with the C# programming language and, finally, it was evaluated by Nielsen's ten principles of usability assessment.

**Results:**

After creating the registry, its data entry search and report functions were tested. Also, in the exploratory evaluation, the highest number of problems related to the principles of system clarity and compatibility between the system and real world was identified.

**Conclusion:**

The web-based DIR created in C# programming language has the ability to gather data, provide report with different access levels, and send text messages to patients for follow-up. This tool enables physicians to quickly identify the components of dental implant. The web-based DIR also provides support for health research, quality assessment, and dental performance assessment.

## 1. Introduction

Dental implant is an alternative solution for patients who are dissatisfied with traditional methods and dentures [1]. About 18 million dental implants are sold annually worldwide, and this figure is projected to grow by 23% by 2026 [2, 3]. Despite advantages such as high success rate, low sensitivity, and bone regeneration, dental implants have biological or mechanical complications [4–6]. Biological complications are divided into two categories of mucositis and peri-implantitis [7]. Early diagnosis of mucositis and peri-implantitis increases the success of treatment [4, 7–9]. On the other hand, damage to the components and structure of dental implants, which is referred to as mechanical complications, leads to loss of implant and increases the number of treatment sessions for dental implant repair [10]. Regular visits to the dentist with a preventive care approach are necessary to control any complications [11, 12]. Identifying the dental implant system is often a challenge for dentists due to the vast variety of implant systems and the long time it takes to identify them. Therefore, it is necessary to store data related to the characteristics of dental implants on a database [13, 14]. In Finland, a national Dental Implant Registry (DIR) system has been in place since 1994 [15]. In Sweden, such system has been in use since 2009 [7]. Registries are a powerful tool for collecting and providing valid data for clinical research, monitoring and evaluating the quality of clinical care, and increasing patient safety [2, 16, 17]. DIR system can improve the safety, quality, and efficiency of dental implants. It does that by allowing patient participation and regular clinical follow-up as well as identifying dysfunctional implants and side effects in the early stages of implant [2, 18]. Lack of reliable and accurate data on dental implants, in addition to imposing high costs on governments and industry, can cause high level of anxiety in patients [19]. In the 4th International Conference on Dental Implants, which was held in July 2018 in Isfahan, statistics of more than 500,000 units of implants per year in Iran were presented [20]. Currently, in Iran, information related to dental implants is collected by paper file [21], and there is no accurate and coherent information about the total number of implants used in the country. Obviously, a web-based DIR will accelerate and facilitate transparent and accurate access to coherent information with regular patient follow-up and better clinical outcomes. It also provides a real-world picture for patients, dentists, manufacturers, and clients and also improves the quality of services for decision-makers and other stakeholders. The present study was an attempt to design and evaluate a DIR system at the Dental Implant Research Center of Tehran University of Medical Sciences. Therefore, the main question of present study is how to design and evaluate a web-based DIR system. Also, the more specific questions include the following:

- What are the objectives of web-based DIR in the Dental Implant Research Center of Tehran University of Medical Sciences?
- How and through what steps is the web-based DIR designed?
- How is the web-based DIR evaluated and what results will be obtained?

## 2. Materials and Methods

This research was conducted in three phases, descriptive-analytical phase, developmental phase, and evaluation phase, to answer the research questions.

### 2.1. Descriptive-Analytical Phase

A systematic review was performed in the descriptive-analytical phase to identify the objectives and data elements [22]. After that, the objectives were classified with the help of two experienced dentists. The different components of the objectives included regulatory, managerial, research, clinical, and epidemiological objectives. Finally, a questionnaire was used by experts to determine the objectives of DIR and also to identify unforeseen needs of the registry. The study population included oral and maxillofacial surgeons, periodontists, and prosthodontists at Dental Implant Research Center of the School of Dentistry, Tehran University of Medical Sciences. In this study, census approach (including all eligible individuals in a quarterly period) was used for sampling. The questionnaire was based on the five-option Likert's scale, so that the most insignificant objective of the registry had a score of one and the most significant objective of the registry had a score of five. An empty space was also considered for the objectives proposed by the dentists. After content validation (CVR) and approval of the research team, the questionnaire was sent to dentists and their opinions were collected. To determine the content validity of the tool at this stage, the Lawshe table was used. For this purpose, first all questions whose CVR value was less than the acceptable level were removed from the questionnaire. Then, the mean CVR values of the accepted questions were considered as the content validity of the tool. In the present study, according to the numerical value of CVR for each question, which was 0.59 for 11 people (4 oral and maxillofacial surgeons, 4 periodontists, and 3 prosthodontists), the number of data elements was removed in each questionnaire. Then, the questionnaire was sent to the research samples (55 people), but only 27 of them completed the questionnaire due to busy schedule and lack of collaboration. To analyze the data, the obtained scores were entered into SPSS software and the mid-point of the measured interval or mid-point response to each item was calculated. In this way, the items that had the mid-point value of 0–2.5 were removed. This was also considered as the inclusion criterion for entry of items to the registry at the third quarter (3.75–5) onwards. The obtained results were used in the next stage of the research.

### 2.2. Developmental Phase

The development phase was carried out in two stages. In the first stage, conceptual models, applications of scenario tables, diagrams, sequences, and activities were designed and plotted by Edraw Max 7.9 software based on the results of previous stage. This was also done by observing the activities and holding symposiums with users and relevant experts. Completeness, support of unified modeling language (UML), ease of use, and possibility of providing output in PDF and JPG files were the reasons for choosing Edraw Max 7.9 software. To confirm the prepared model, the diagrams were provided to the research team and their corrective comments and suggestions were considered and, finally, the desired changes were applied to the diagrams. The second step involved determining the hardware and software requirements needed to create the registry. The registry of dental implants is a web-based system that uses ASP.net framework. The registry was designed in visual studio environment using C# programming language. The SQL server database was also used to store the data. Also, the NET 4.5 technology platform was used and Cristal Report was considered for preprint reports and output. Multilayer architecture was also used to design the registry.

### 2.3. Evaluation Phase

The evaluation phase included testing the registry performance and evaluating its usability. In the first stage, the system performance was evaluated by the researcher by entering data, searching and reporting data of 58 dental implant files, and, finally, testing the mentioned functions. The study population consisted of the data of patients who had attended the Dental Implant Research Center in 2019. In the next step, the system was evaluated by using Nielsen's ten principles of usability assessment. The study population consisted of health information management specialists (2 people) and medical informatics (3 people) who had been selected by convenience sampling method. Each of the evaluators independently examined the different parts of the registry to make sure it complies with Nielsen's ten principles. After identifying the problems by each assessor, the duplicates were removed. Then, the severity of identified problems was ranked from 0 to 4 by the evaluators based on Nielsen's ranking [23] and the data were analyzed by SPSS software version 21.

## 3. Research Findings

The main findings of this research have been divided into three main phases of descriptive-analytical phase, developmental phase, and evaluation phase.

### 3.1. Descriptive-Analytical Phase

This phase included identifying and determining the objectives of DIR.  Table 1 shows the list of registry objectives along with the CVR and midpoint values obtained for each objective. Reporting the treatment side effects (4.81), following and monitoring diseases that affect treatment outcome (4.66), and monitoring survival/success of implants (4.55) were among the objectives that had the highest midpoint.

### 3.2. Development Phase

#### 3.2.1. Design of the DIR Conceptual Model

**Figure 1:**
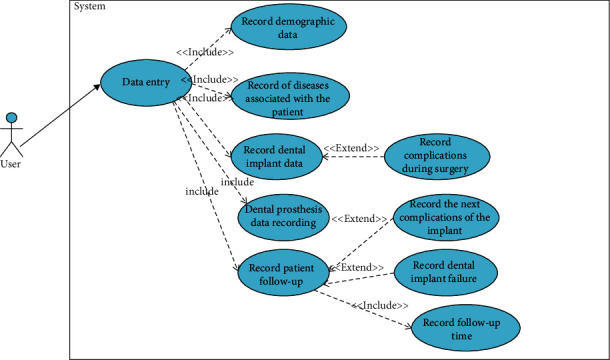
Diagram used to record data in the DIR.

An example of diagrams and scenario table with explanations are presented in this section. The diagram used to record data in the DIR is shown in Figure 1.  Table 2 shows the examples of scenario related to entry of implant data in the DIR.

#### 3.2.2. Development of the DIR

**Figure 2:**
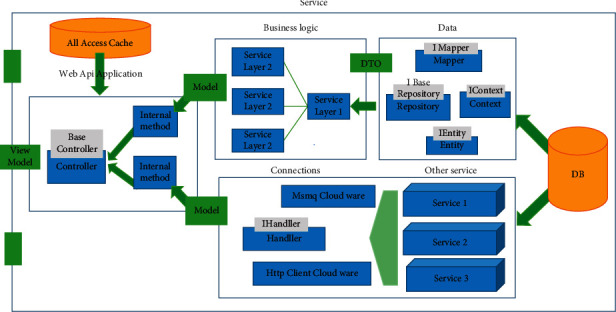
General architecture of dental implant registry.

In order to create a web-based DIR, the service and data-based architecture was used. In this model, the system design starts from the data model, and the other layers are designed depending on the data model. The general architecture of DIR is shown in Figure 2.

**Figure 3:**
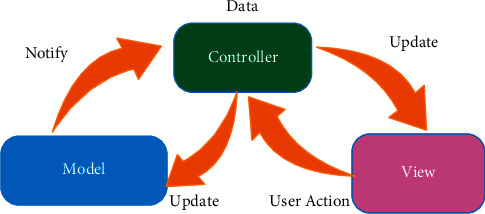
Architecture of the inner layer of the DIR.

This architecture consists of 6 parts:

(1) Database: SQL is used in the database layer. Here, all data related to users, maps, patient records, and referrals are stored.
(2) Data: This is the layer in which software communicates with the database and receives and transmits data. This layer consists of 4 parts:
  - Connect to management
  - Define data model
  - Connect the data model in the software and the data model in the database
  - Read, create, update, and delete functions from the database
(3) DB: this is where the most used data is stored.
(4) Business logic: in this section, the connection with the database layer is created to eliminate the complexities, processes, and logic of the software.
(5) Other services and connections: in this layer, the software communicates with other services that are available in the comprehensive software system and implements the needs and processes that are dependent on other services according to the needs.
(6) Web API application: this is the final layer and place where the software communicates with the outside environment. Here, depending on the client's needs, it uses a combination of two layers and provides the desired model that the client requests by using the business logic layer and communicating with other services. The architecture of the inner layer of the system consists of three layers: model, observer, and controller (Figure 3).

Model layer: this layer is one of the main sections in the three-layer architecture. The main task of this section is to communicate with the “phpMyAdmin database,” “SQL server,” and other sections, such as retrieving information from database tables or reading information from them. This section is also responsible for checking the data accuracy.

Observer layer: this section is actually the section that the user is dealing with, and its main task is to take information from the model and controller layers and display them in the user side section.

**Figure 4:**
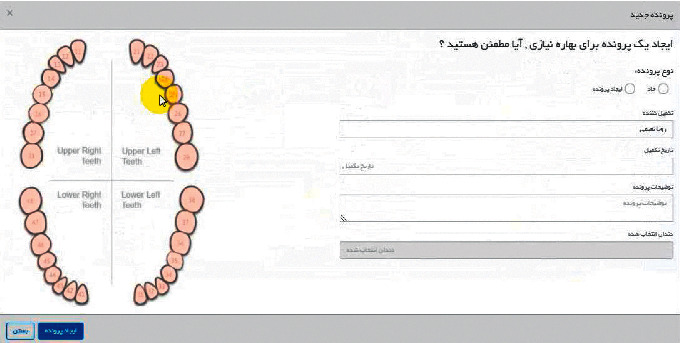
Step of determining the location of tooth in order to enter the data in the DIR.

Controller layer: as the name implies, this section is the controller and the interface between the two model and observer layers and its main task is to establish communication between the user and the server side part.  Figure 4 shows part of the scenario of recording dental implant data in DIR.

### 3.3. Evaluation Phase

#### 3.3.1. Results of Function Test for the DIR

According to the reviewed files, the highest number of dental implants was related to lower left 37 and lower left 36 with 15.5%. This tooth numbering is based on FDI standard. The results of present study showed that 53.4% of women and 46.6% of men underwent dental implant surgery in 2019. Also, 5 patients (8.6%) had cardiovascular disease, 6 patients (10.3%) had gastrointestinal disease, and 4 patients (6.9%) had intestinal disease. Moreover, 7 patients (12.1%) were smokers: 5 patients (8.6%) smoked 1–5 cigarettes per day and 2 (3.4%) smoked 6–15 cigarettes per day. Based on the results of data analysis, 77.6% of the diameter of dental implant system used was 4.1.

#### 3.3.2. Usability Assessment of the DIR

The evaluation identified 90 problems in different parts of the system. After eliminating the repetitive problem, 50 problems were evaluated by the evaluators in terms of severity based on three characteristics of repetition frequency, effect of problem on the system, and durability and stability of the problem. Each of the evaluators explored 4 (8%), 24 (48%), 7 (14%), 5 (10%), and 10 (20%) problems independently. The results of exploratory evaluation of the DIR showed that, among the identified problems, the highest number of problems was related to the principle of system clarity (28%) and then the principle of concordance between the system and the real world (18%). The lowest number of problems was related to the principles of diagnosis instead of reminder (2%) and flexibility and efficiency of use (2%). From the all identified problems, only the principle of error prevention with an average problem severity of 3.04 was identified as the major problem of the system. Problems identified by the evaluators were minor problems that had low priority for correction (Table 3).

## 4. Discussion

In this study, according to the panel of experts, the main objectives of DIR included report of treatment side effects, follow-up and monitoring of the diseases affecting the treatment outcome, and monitoring of the survival/success of implants. The first step in designing a registry is to set objectives. A registry should be designed based on predetermined objectives [24]. Registry objectives affect its domain, design, structure, data collection process, and minimum dataset [24–26]. If the registry development team feels that its objectives are being met, its motivation to work will increase [27]. By setting registry objectives and defining the processes of collecting, processing, and distributing information in a transparent manner, the registry objectives will be achieved and it will be possible to follow up, prevent, and control the complications of dental implants. Therefore, in the first step of the present study, the DIR objectives were determined. Analysis of healthcare systems is complex and difficult, and the UML is used to analyze healthcare systems [28]. Successful UML modeling requires the use of appropriate information and diagrams to achieve objectives [29]. According to Kendall, UML provides the design team with more complete information through documentation and diagrams for system visualization and final production. For the success of UML, complete and accurate system model should be provided to design team, which facilitates better understanding and coordination between the research and IT teams with regard to system needs [30]. In the present study, UML diagrams were used for its benefits, so that a suitable system can be considered according to the anatomy of teeth to meet the needs of dentists. Ko et al. used Java, Apache web server, MySQL database, and Cocoon framework to design a web-based portal for diabetes [31]. Norman Faden et al. developed an open-source diabetes care with tools from MySQL Workbench, Java Development Kit, Apache Tomcat, and NetBeans [32]. Around the world, several countries such as United States [33], Sweden [34], Canada [35], and Australia [36] have taken steps to create and use DIR registry. In Germany, software called impDAT has been introduced since 2008 to document, manage, and evaluate dental procedures in the form of software and network [37]. Given the variety of tools and technologies used to create a web-based system, the use of a particular language and framework or technology depends on the available purpose, application, features, and capabilities [38]. The output of the present study is a web-based registry for dental implant, the main features of which include structured data entry, reporting with defined access levels, and sending text messages to patients. Web-based software facilitates communication and interaction between healthcare users and makes it possible to record patient data from several centers simultaneously in a uniform and integrated manner [39]. Aqle acknowledged that Nielsen's assessment is effective in identifying problems, and the possibility of ignoring problems is minimized in this approach [40]. According to Zhang, Nielsen's exploratory evaluation is an easy, efficient, cost-effective, and useful way to identify problems and their severity and also classify and prioritize problems before routine use of the system [41–43]. The usability of system in performing tasks helps users to do their work quickly and with minimal effort [44]. Usability is directly related to error, productivity, and user satisfaction [45], so that the low usability of system reduces the use of information by users [44]. The evaluation of DIR showed that the principle of “error prevention” had the highest mean severity, and the severity of problem was in the category of major problems. Also, there was a high priority for error correction before routine use of the system. The results of this study can provide dentists, policymakers, Ministry of Health, and other stakeholders a good basis for following up and controlling complications of dental implants and promoting oral health. It is also a good platform for designing and conducting valid and robust studies that require a registry, such as nested case-control study, case-cohort study, or subsample trials. The high priority of the present study in the Dental Implant Research Center of Tehran University of Medical Sciences, the participation of experienced and interested dentists in the development of registry at the Dental Implant Research Center of Tehran University of Medical Sciences, the support of Dental Implant Research Center of Tehran University of Medical Sciences by accepting the cost of registry design, and the use of maximum number of evaluators to perform exploratory evaluation are among the strengths of this study. However, just considering the opinions of dentists at the School of Dentistry of Tehran University of Medical Sciences is one of the limitations of present study. Also, in this study, the system was not evaluated by end-users, as it has been suggested by other studies. When we obtain the results of piloting the system, the objectives and performance of the system can be widely studied in universities across the country. This research has received the ethics permission with the code IR.TUMS.SPH.REC.1397.295 from the Research Ethics Committee of Tehran University of Medical Sciences.

## 5. Conclusion

There is no accurate information on the total number of dental implants in Iran. The lack of a DIR poses a challenge to clinical research, evaluation of treatment outcomes, and monitoring of the quality of care. The web-based DIR, created in C# programming language, is an appropriate tool that provides easy, accurate, and quick access to patient information.

The main features of web-based DIR include structured data entry, reporting with defined access levels, and sending text messages to patients. This system enables dentists to quickly identify implant components (type, diameter, and length), provide effective treatment and follow the treatment results, and control treatment complications. Web-based DIR, by collecting clinical data and providing regular follow-up of patients, facilitates health research, quality assessment, and evaluation of dental implant performance.

## Figures and Tables

**Table 1:** Objectives of DIR along with CVR and midpoint values.

Objectives		CVR	Accepted or deleted	Mid-point
Observational	Report the treatment's side effects	1	√	4.81
	Track the positive or negative process of dental implant treatment	0.66	√	4.22
	Follow the treatment's results based on different brands	0.66	√	4.29
	Monitor the survival/success of implants	0.83	√	4.55
	Comparison of the survival/success rates of implants	0.83	√	4.29

Managerial	Recognize the types of complications and risks after dental implant treatment	0.66	√	4.18
	Evaluate biological and mechanical complications	0.66	√	4.03
	Prevention and reduction of implant complications and complaints	0.66	√	4.22
	24-Hour access to patient information and records, 7 days a week	0.66	√	4.11
	Radiographic documentation	0.83	√	4.44
	Improve communication between patients and therapists	0.16	^*∗*^	—
	Improve patient satisfaction	0.5	^*∗*^	—

Research	Epidemiological research	0.66	^*∗*^	4.29
	Provide information for research	0.83	√	4.4
	Participate in multicenter studies	0.83	√	4.37
	Provide action-based evidence	1	√	4.48
	Predict implant failure	0.33	√	—
	Assist in the development of national guidelines	0.5	^*∗*^	—

Clinical	Evaluation of clinical advantages of different types of dental implant systems	0.66	^*∗*^	4.29
	Clinical evaluation of dental implant systems	0.83	√	4.4
	Investigate the effect of age, sex, and place of implant treatment on implant failure	0.83	√	4.37
	Follow-up and monitoring of diseases affecting the outcome of treatment	1	√	4.66
	Comparison of different treatments or methods in implant treatment	0.66	√	4.37
	Regular follow-up and monitoring of patients	0.83	√	4.62

Epidemiological	Prevalence and complications of dental implants	0.66	√	4.29
	Evaluation of risk factors in peri-implantitis	0.83	√	4.4

√: acceptance of objective; ^*∗*^: rejection of objective; —: not earning CVR points and elimination of objective.

**Table 2:** Scenario related to the recording of dental implant data in DIR.

Name of application	Dental implant entry	
Code	UC3.3	
Scenario	Recording of dental implant data	
Brief description	Through this use case, the dentist records the patient's dental implant data in the system	
Care provider	Dentist	
Prerequisites	It is necessary for the user to login to the system	
Prerequisites	The patient's dental implant home screen is displayed	

Activity process	Care provider	System manager
	1: the dentist selects the location of dental implant from the tooth image 2: the dentist records the patient's dental implant data in the relevant form 3: the dentist selects the confirmation option 4: the dentist completes information about complications and problems during surgery	1: the system shows the user the page related to the dental implant, which contains the tooth image and FDI numbering 1–2: it is possible to provide a list of implanted teeth by date in the system 1–3. The accuracy of data is checked 1–4: the dentist answers the item: “Did any complications occur during the surgery?” If the answer is yes, the dentist will be directed to the page of complications and problems during surgery; if the answer is no, the information will be saved. 1–5: the accuracy of data is checked in the system 2–5: data are recorded in the system

Exceptional situations	1: if the dentist enters the mandatory data in the fields incompletely or the data is not entered in a specific format, the system will give him an error message in red and will prevent the storage of other information until it is corrected 2: if the user presses the cancel button at any stage of the process, the system will exit the data registration form without any changes 3: if the system has problems in storing information, the necessary message will be shown to the dentist and it will be removed from the data registration form without any changes	

**Table 3:** Number of usability problems identified based on Nielsen's evaluation principles.

No.	Principles of evaluation	Number of problems (%)	Severity	Number of problems identified					Severity of problem
				Evaluator 1	Evaluator 2	Evaluator 3	Evaluator 4	Evaluator 5	
1	System clarity	14 (20)	1.8	4	5	1	3	1	Small
2	Concordance between the system and the real world	9 (18)	1.7	0	7	0	0	2	Small
3	Freedom of user and control of the system	4 (20)	1.95	0	1	1	1	1	Small
4	Observance of uniformity and standards	3 (20)	1.4	0	1	0	1	1	Minor
5	Help diagnose, identify, and correct errors	6 (12)	2.3	0	3	1	0	2	Small
6	Error prevention	5 (10)	3.04	0	3	1	0	1	Big
7	Recognition instead of reminders	1 (20)	1.8	0	1	0	0	0	Small
8	Flexibility and efficiency of use	1 (20)	2	0	1	0	0	0	Small
9	Aesthetic aspects and simple design	5 (10)	1.4	0	1	3	0	1	Minor
10	Guidance and documentation	2 (4)	2.4	0	1	0	0	1	Small
Total		50 (100)	1.8	4	24	7	5	10	Small

## Data Availability

Information on conceptual modeling, CVR, mid-point, and evaluation phase of this paper will be provided if requested and submitted to the access committee and approved.

## References

[1] Sun T.-M., Lan T.-H., Pan C.-Y., Lee H.-E. (2018). Dental implant navigation system guide the surgery future. *The Kaohsiung Journal of Medical Sciences*.

[2] Klinge B., Lundström M., Rosén M., Bertl K., Klinge A., Stavropoulos A. (2018). Dental Implant Quality Register-A possible tool to further improve implant treatment and outcome. *Clinical Oral Implants Research*.

[3] Elani H. W., Starr J. R., Da Silva J. D., Gallucci G. O. (2018). Trends in dental implant use in the U.S., 1999–2016, and projections to 2026. *Journal of Dental Research*.

[4] Findler M., Chackartchi T., Regev E. (2014). Dental implants in patients at high risk for infective endocarditis: a preliminary study. *International Journal of Oral and Maxillofacial Surgery*.

[5] Gurgel B. C. d. V., Montenegro S. C. L., Dantas P. M. C., Pascoal A. L. d. B., Lima K. C., Calderon P. d. S. (2017). Frequency of peri-implant diseases and associated factors. *Clinical Oral Implants Research*.

[6] Cicciù M., Fiorillo L., Herford A. S. (2019). Bioactive titanium surfaces: interactions of eukaryotic and prokaryotic cells of nano devices applied to dental practice. *Biomedicines*.

[7] Derks J., Schaller D., Håkansson J., Wennström J. L., Tomasi C., Berglundh T. (2016). Effectiveness of implant therapy analyzed in a Swedish population. *Journal of Dental Research*.

[8] Cervino G., Fiorillo L., Iannello G., Santonocito D., Risitano G., Cicciù M. (2019). Sandblasted and acid etched titanium dental implant surfaces systematic review and confocal microscopy evaluation. *Materials*.

[9] Cervino G., Meto A., Fiorillo L. (2021). Surface treatment of the dental implant with hyaluronic acid: an overview of recent data. *International Journal of Environmental Research and Public Health*.

[10] Brägger U., Karoussis I., Persson R., Pjetursson B., Salvi G., Lang N. P. (2005). Technical and biological complications/failures with single crowns and fixed partial dentures on implants: a 10 year prospective cohort study. *Clinical Oral Implants Research*.

[11] Tonetti M. S. (2015). Peri-implantitis: etiology, pathogenesis, prevention, and therapy. *Dental Implant Complications: Etiology, Prevention, and Treatment*.

[12] Tarawali K. (2015). Maintenance and monitoring of dental implants in general dental practice. *Dental Update*.

[13] Derks J., Håkansson J., Wennström J. L., Tomasi C., Larsson M., Berglundh T. (2015). Effectiveness of implant therapy analyzed in a Swedish population. *Journal of Dental Research*.

[14] Michelinakis G., Sharrock A., Barclay C. W. (2006). Identification of dental implants through the use of implant recognition software (IRS). *International Dental Journal*.

[15] Antalainen A. K., Helminen M., Forss H., Sándor G. K., Wolff J. (2013). Assessment of removed dental implants in Finland from 1994 to 2012. *The International Journal of Oral & Maxillofacial Implants*.

[16] von Bültzingslöwen I., Östholm H., Gahnberg L., Ericson D., Wennström J. L., Paulander J. (2019). Swedish quality registry for caries and periodontal diseases–a framework for quality development in dentistry. *International Dental Journal*.

[17] McNeil J. J., Evans S. M., Johnson N. P., Cameron P. A. (2010). Clinical‐quality registries: their role in quality improvement. *Medical Journal of Australia*.

[18] Klinge B., Sanz M., Alcoforado G. (2018). Dental implant register: summary and consensus statements of group 2. The 5th EAO Consensus Conference 2018. *Clinical Oral Implants Research*.

[19] Cooter R. D., Barker S., Carroll S. M. (2015). International importance of robust breast device registries. *Plastic and Reconstructive Surgery*.

[20] https://https://civilica.com/l/9372/.

[21] Naseri R., Mansouri M., Moochani A. (2018). Frequency of dental implants placed in the Dental School of Isfahan University of Medical Sciences based on surgical and prosthetic factors. *Journal of Isfahan Dental School*.

[22] Naemi R., Barikani H. R., Shahmoradi L. (2021). Dental implant quality registries and databases: a systematic review. *Journal of Education and Health Promotion*.

[23] Aqle A., Khowaja K., Al-Thani D. Accessibility or usability of InteractSE? A heuristic based approach to evaluate proposed search engine for the visually impaired users.

[24] Gliklich R. E., Dreyer N. A., Leavy M. B. (2010). *Registries for Evaluating Patient Outcomes*.

[25] Viviani L., Zolin A., Mehta A., Olesen H. V. (2014). The European Cystic Fibrosis Society Patient Registry: valuable lessons learned on how to sustain a disease registry. *Orphanet Journal of Rare Diseases*.

[26] Rankin J., Best K. (2014). Disease registers in england. *Paediatrics and Child Health*.

[27] Evatt B. (2005). *World Federation of Hemophilia Guide to Developing a National Patient Registry*.

[28] Vasilakis C., Lecznarowicz D., Lee C. (2009). Developing model requirements for patient flow simulation studies using the Unified Modelling Language (UML). *Journal of Simulation*.

[29] van der Maas A. A. F., Ter Hofstede A. H. M., Ten Hoopen A. J. (2001). Requirements for medical modeling languages. *Journal of the American Medical Informatics Association*.

[30] Kendall K. E., Kendall J. E., Kendall E. J., Kendall J. A. (2002). *Systems Analysis and Design*.

[31] Ko G. T., So W. Y., Tong P. C. (2010). From design to implementation--the Joint Asia Diabetes Evaluation (JADE) program: a descriptive report of an electronic web-based diabetes management program. *BMC Medical Informatics and Decision Making*.

[32] Norman McFadden B. D., Hoyt R., Snider D. (2017). Development of a web-based registry to support diabetes care in free medical clinics. *Perspectives in Health Information Management*.

[33] Weyant R. J., Burt B. A. (1993). An assessment of survival rates and within-patient clustering of failures for endosseous oral implants. *Journal of Dental Research*.

[34] Jemt T. (2018). Implant survival in the edentulous jaw-30 years of experience. Part I: a retro-prospective multivariate regression analysis of overall implant failure in 4,585 consecutively treated arches. *International Journal of Prosthodontics*.

[35] Arlin M. L. (2002). Analysis of 435 Screw-Vent dental implants placed in 161 patients: software enhancement of clinical evaluation. *Implant Dentistry*.

[36] Guo Q., Lalji R., Le A. (2015). Survival rates and complication types for single implants provided at the Melbourne Dental School. *Australian Dental Journal*.

[37] Krebs M., Schmenger K., Neumann K., Weigl P., Moser W., Nentwig G. H. (2015). Long‐term evaluation of ANKYLOS® dental implants, part I: 20 year life table analysis of a longitudinal study of more than 12,500 implants. *Clinical Implant Dentistry and Related Research*.

[38] Vitolo C., Elkhatib Y., Reusser D., Macleod C. J. A., Buytaert W. (2015). Web technologies for environmental big data. *Environmental Modelling & Software*.

[39] Zakerabasali S., Kadivar M., Safdari R. (2020). Development and validation of the neonatal abstinence syndrome minimum data set (NAS-MDS): a systematic review, focus group discussion, and delphi technique. *Journal of Maternal-Fetal and Neonatal Medicine*.

[40] Aqle A., Khowaja K., Al-Thani D. (2020). Preliminary evaluation of interactive search engine interface for visually impaired users. *IEEE Access*.

[41] Zhang J., Johnson T. R., Patel V. L., Paige D. L., Kubose T. (2003). Using usability heuristics to evaluate patient safety of medical devices. *Journal of Biomedical Informatics*.

[42] Graham M. J., Kubose T. K., Jordan D., Zhang J., Johnson T. R., Patel V. L. (2004). Heuristic evaluation of infusion pumps: implications for patient safety in Intensive Care Units. *International Journal of Medical Informatics*.

[43] Scandurra I., Hagglund M., Koch S., Lind M. (2008). Usability laboratory test of a novel mobile homecare application with experienced home help service staff. *The Open Medical Informatics Journal*.

[44] Rangraz Jeddi F., Nabovati E., Bigham R., Khajouei R. (2020). Usability evaluation of a comprehensive national health information system: relationship of quality components to users’ characteristics. *International Journal of Medical Informatics*.

[45] Ahmadian L., Salehi F., Abedinzadeh A., Khatibi F. (2017). Usability evaluation of a radiology information system. *Journal of Health Administration (JHA)*.

